# Mini bat organs reveal hidden viral threats

**DOI:** 10.1002/ctm2.70454

**Published:** 2025-08-17

**Authors:** Hyunjoon Kim, Bon‐Kyoung Koo, Young Ki Choi

**Affiliations:** ^1^ Center for Study of Emerging and Re‐emerging Viruses Korea Virus Research Institute, Institute for Basic Science Daejeon Republic of Korea; ^2^ Centre for Genome Engineering Institute for Basic Science Daejeon Republic of Korea

## 1 WHY STUDY BATS?

Bats are extraordinary animals. Beyond their vital ecological roles in pollination, seed dispersal, and insect control,^1^ they are also key reservoirs for a wide array of zoonotic viruses.^2^ But what makes bats so incredible to be major mammalian reservoirs for a number of deadly viruses to animals and humans, such as the Rabies virus, Ebola virus, Nipah virus, as well as coronaviruses responsible for severe acute respiratory syndrome (SARS), Middle East respiratory syndrome (MERS) and coronavirus disease 2019 (COVID‐19)?^2^, ^3^

First, their ability to fly allows for wide geographic dispersal, enhancing opportunities for viral spread across ecosystems.^1^, ^2^ Second, bats exhibit remarkable species diversity, over 1400 species, providing vast evolutionary niches for viruses.^2^ Notably, bats harbour a greater number of viruses per species than any other mammalian order.^2^, ^4^ Third, bats have unique immune features, such as robust antiviral interferon responses and dampened inflammatory responses during viral infections,^2^ allowing them to tolerate viruses that are lethal to other animals and humans without getting sick themselves. These features have placed bats at the centre of virome surveillance and pandemic preparedness efforts, yet effective experimental systems for studying bat‐virus interactions remain limited.

## 2 KEY RESEARCH GAPS IN PREVIOUS BAT ORGANOID MODELS

To effectively study emerging and re‐emerging bat‐borne viruses, physiologically relevant in vitro bat model systems are required for the isolation and functional characterisation of these pathogens. Although some bat epithelial organoids have been developed from respiratory and enteric tissue,^5^, ^6^, ^7^ existing models possess some key limitations that constrain their utility in virome surveillance efforts. First, most are derived from tropical fruit bats, with little representation of insectivorous bats, especially the Vespertilionidae family (vesper bats), the most diverse and globally widespread lineage, which is predominant in temperate regions, including Korea, Japan and China. Second, most available models are restricted to a single organ type, such as the respiratory or enteric epithelium, thereby limiting comprehensive analysis of viral tissue tropism across major transmission routes. Third, these models are not integrated into a functional pipeline for virus isolation, characterisation and antiviral screening.

To address these gaps, we have developed a suite of multi‐species, multi‐organ bat epithelial organoid models.^8^ These include organoids representing respiratory (tracheal and bronchoalveolar), enteric and renal tissues, derived from multiple insectivorous bat species, specifically, four vesper bat species and one horseshoe bat. Although all bat species were captured in South Korea, these species not only reflect the local chiropteran diversity of Northeast Asia but also represent members of the most diverse and widely distributed bat family globally.^9^

**FIGURE 1:**
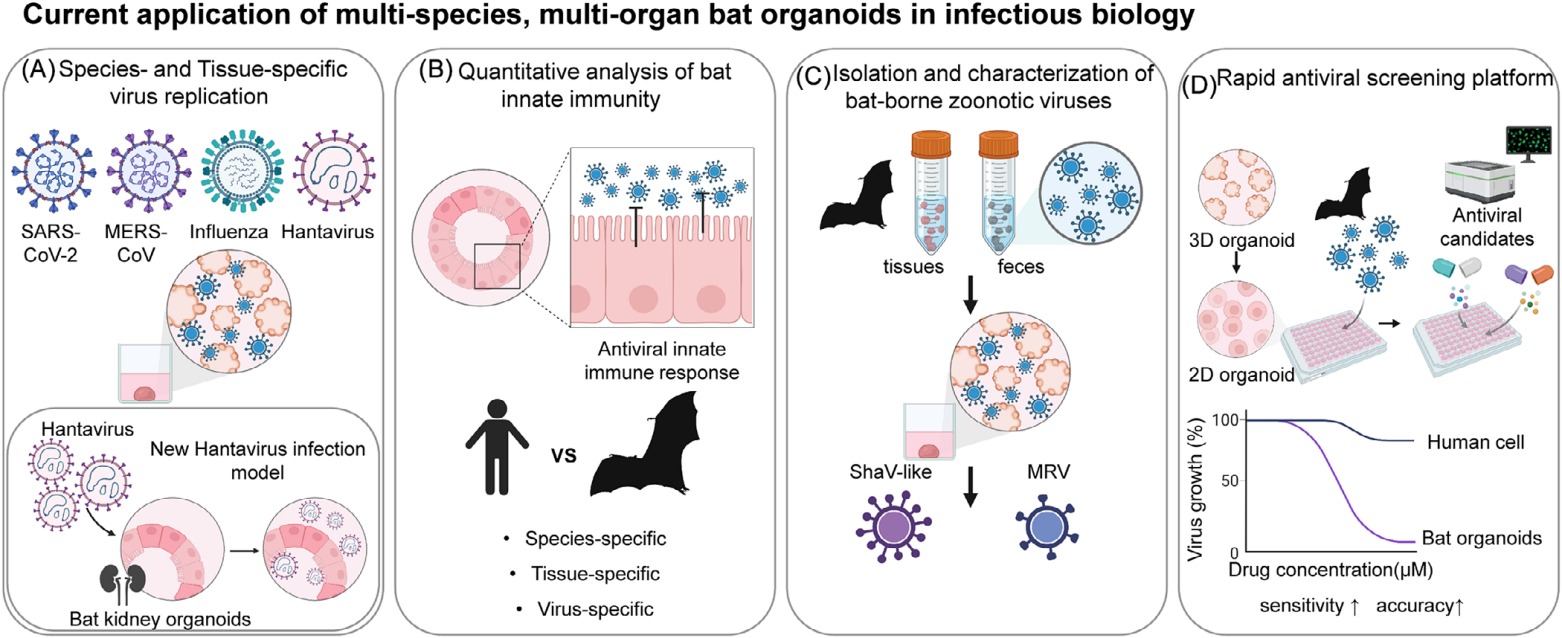
Current application of the multi‐species, multi‐organ bat organoid platform. (A–D) The multi‐species, multi‐organ bat organoid platform can be utilised to dissect species‐ and tissue‐specific virus replication (A), quantitatively analyse bat innate immunity (B), isolate and characterise emerging and re‐emerging bat‐borne viruses (C) and rapidly screen potential antiviral drugs (D).

Notably, Kellner et al. recently established respiratory and intestinal organoids derived from the Egyptian fruit bat (*Rousettus aegyptiacus*), a natural reservoir of Marburg virus, providing a valuable model for studying tissue‐specific viral replication and immune modulation in this species.^10^ While their work represents an important step forward in bat organoid research, the model remains limited to a single frugivorous species from a tropical region. In contrast, our study expands the field significantly by establishing organoids from multiple insectivorous bat species, particularly from temperate zones that are underrepresented in previous work, and by incorporating four distinct epithelial tissue types relevant to major routes of viral entry. Moreover, our platform enables both the isolation of novel bat‐borne viruses and the characterisation of species‐ and tissue‐specific innate immune responses, offering a more comprehensive and scalable system for virome surveillance and translational virology applications (Figure 1).

## 3 APPLICATIONS OF MULTI‐SPECIES, MULTI‐ORGAN BAT ORGANOIDS IN INFECTIOUS BIOLOGY

The multi‐species, multi‐organ bat organoid platform offers a powerful tool to study species‐ and tissue‐specific viral replication dynamics. For example, SARS‐coronavirus 2 (SARS‐CoV‐2) replicated selectively in small intestinal organoids of *Rhinolophus ferrumequinum*—a close relative of the presumed SARS‐CoV/SARS‐CoV‐2 reservoir *R. sinicus*—but not in organoids from vesper bats. MERS‐CoV showed highly species‐specific replication patterns even among closely related Vespertilionidae species. Similarly, Seoul virus, a Hantavirus causing hemorrhagic fever with renal syndrome, exhibited kidney‐specific replication and cytopathic effects, illustrating the platform's utility in dissecting tissue tropism.

Beyond replication, this system enables detailed investigation of species‐, tissue‐ and virus‐specific innate immune responses. Using spatiotemporal transcriptome analyses, we profiled the activation of interferon and antiviral signalling pathways. While cross‐species comparisons require high‐quality genomes, we are generating reference‐grade assemblies for several bat species. As proof‐of‐concept, we profiled interferon‐stimulated gene (ISG) responses to MERS‐CoV, influenza A, Seoul virus, a bat mammalian orthoreovirus isolate, and a Shaan virus‐like paramyxovirus in *R. ferrumequinum* organoids. These revealed distinct virus‐ and tissue‐specific ISG signatures, demonstrating the platform's power in studying innate immunity across the bat virome.

Lastly, our bat organoid system supports efficient virus isolation and characterisation in a physiologically relevant host context. Compared to conventional cell lines, organoids offer more accurate tissue environments, enabling virus recovery that might otherwise fail. They also allow investigation of host‐virus interactions and cell type‐specific susceptibility. Importantly, the platform enables rapid antiviral drug screening, reducing the need for early in vivo testing. Together, these features streamline virome surveillance and response efforts, positioning this system as a valuable tool for identifying and characterising emerging bat‐borne viruses in preparation for future pandemics.

## 4 FUTURE DIRECTIONS

Moving forward, we aim to further advance the multi‐species, multi‐organ bat epithelial organoid platform by incorporating components of the immune system to better recapitulate the complex immune‐epithelial interface. Specifically, we are developing co‐culture systems that integrate immortalised, species‐matched bone marrow–derived macrophages with epithelial organoids, enabling more physiologically relevant modelling of host–virus interactions and innate immune responses. We also plan to broaden the taxonomic and anatomical scope of our platform. This includes generating organoids from additional bat families, rodents (the most diverse mammalian order), and other mammals and birds. These efforts will be supported by parallel genome assembly initiatives, which will facilitate more precise interpretation of host responses and comparative virology across species. Beyond infectious disease applications, we envision establishing an “organoid zoo”, a living biobank of organoids from phylogenetically diverse animals, to explore fundamental biological questions. Such a resource would enable cross‐species investigations into conserved and divergent mechanisms underlying tissue regeneration, ageing and cancer, offering a powerful tool for both evolutionary biology and translational research.

## AUTHOR CONTRIBUTIONS

All authors equally contributed to the writing of the letter.

## CONFLICT OF INTEREST STATEMENT

The authors declare no conflicts of interest.

## ETHICS STATEMENT

This article does not contain any research involving humans or animals.
